# Klotho inversely relates with carotid intima- media thickness in atherosclerotic patients with normal renal function (eGFR ≥60 mL/min/1.73m^2^): a proof-of-concept study

**DOI:** 10.3389/fendo.2023.1146012

**Published:** 2023-05-19

**Authors:** Javier Donate-Correa, Ernesto Martín-Núñez, Alberto Martin-Olivera, Carmen Mora-Fernández, Víctor G. Tagua, Carla M. Ferri, Ángel López-Castillo, Alejandro Delgado-Molinos, Victoria Castro López-Tarruella, Miguel A. Arévalo-Gómez, Nayra Pérez-Delgado, Ainhoa González-Luis, Juan F. Navarro-González

**Affiliations:** ^1^ Unidad de Investigación, Hospital Universitario Nuestra Señora de Candelaria (HUNSC), Santa Cruz de Tenerife, Spain; ^2^ GEENDIAB (Grupo Español para el estudio de la Nefropatía Diabética), Sociedad Española de Nefrología, Santander, Spain; ^3^ Instituto de Tecnologías Biomédicas, Universidad de La Laguna, Santa Cruz de Tenerife, Spain; ^4^ RICORS2040 (Red de Investigación Renal-RD21/0005/0013), Instituto de Salud Carlos III, Madrid, Spain; ^5^ Área de Medicina Preventiva y Salud Pública, Universidad de La Laguna, San Cristóbal de La Laguna, Spain; ^6^ Escuela de Doctorado y Estudios de Posgrado, Universidad de La Laguna, San Cristóbal de La Laguna, Spain; ^7^ Vascular Surgery Service, HUNSC, Santa Cruz de Tenerife, Spain; ^8^ Servicio de Anatomía Patológica, HUNSC, Santa Cruz de Tenerife, Spain; ^9^ Departamento de Anatomía e Histología Humana, Universidad de Salamanca, Salamanca, Spain; ^10^ Servicio de Análisis Clínicos, HUNSC, Santa Cruz de Tenerife, Spain; ^11^ Servicio de Nefrología, HUNSC, Santa Cruz de Tenerife, Spain

**Keywords:** Klotho, carotid, intima and media thickness, gene expression data, serum, immunohistochemistry

## Abstract

**Introduction:**

Klotho protein is predominantly expressed in the kidneys and has also been detected in vascular tissue and peripheral blood circulating cells to a lesser extent. Carotid artery intima-media thickness (CIMT) burden, a marker of subclinical atherosclerosis, has been associated with reductions in circulating Klotho levels in chronic kidney disease patients, who show reduced levels of this protein at all stages of the disease. However, the contribution of serum Klotho and its expression levels in peripheral blood circulating cells and in the carotid artery wall on the CIMT in the absence of kidney impairment has not yet been evaluated.

**Methods:**

We conducted a single-center study in 35 atherosclerotic patients with preserved kidney function (eGFR≥60 mL/min/1.73m2) subjected to elective carotid surgery. Serum levels of Klotho and cytokines TNFa, IL6 and IL10 were determined by ELISA and transcripts encoding for Klotho (KL), TNF, IL6 and IL10 from vascular segments were measured by qRT-PCR. Klotho protein expression in the intima-media and adventitia areas was analyzed using immunohistochemistry.

**Results:**

APatients with higher values of CIMT showed reduced Klotho levels in serum (430.8 [357.7-592.9] vs. 667.8 [632.5-712.9] pg/mL; p<0.001), mRNA expression in blood circulating cells and carotid artery wall (2.92 [2.06-4.8] vs. 3.69 [2.42-7.13] log.a.u., p=0.015; 0.41 [0.16-0.59] vs. 0.79 [0.37-1.4] log.a.u., p=0.013, respectively) and immunoreactivity in the intimal-medial area of the carotids (4.23 [4.15-4.27] vs. 4.49 [4.28-4.63] log µm2 p=0.008). CIMT was inversely related with Klotho levels in serum (r= -0.717, p<0.001), blood mRNA expression (r=-0.426, p=0.011), and with carotid artery mRNA and immunoreactivity levels (r= -0.45, p=0.07; r= -0.455, p= 0.006, respectively). Multivariate analysis showed that serum Klotho, together with the gene expression levels of tumor necrosis factor TNFa in blood circulating cells, were independent determinants of CIMT values (adjusted R2 = 0.593, p<0.001).

**Discussion:**

The results of this study in subjects with eGFR≥60mL/min/1.73m2 show that patients with carotid artery atherosclerosis and higher values of CIMT present reduced soluble Klotho levels, as well as decreased KL mRNA expression in peripheral blood circulating cells and Klotho protein levels in the intima-media of the carotid artery wall.

## Introduction

Atherosclerosis is the underlying process of most cardiovascular events, the main cause of death in middle-aged and elderly people (1). This process is thought to be a chronic inflammatory disease initiated and perpetuated by a variety of cardiovascular risk factors (2). Klotho protein, an important regulator of mineral metabolism (3), has been reported to exert a protective anti-inflammatory role in the pathogenesis of atherosclerosis in both clinical and preclinical studies (4–12).Klotho is a single-pass transmembrane protein predominantly expressed in the kidneys and, at lesser extent, it has also been detected in the vasculature, and in peripheral blood circulating cells (PBCCs) (13–15). A circulating soluble form of Klotho is also generated by proteolytic cleavage of the extracellular domain of the membranous protein. This shedding is operated by 2 metalloproteinases of the A disintegrin and metalloproteinase domain containing protein (ADAM) family (ADAM10 and ADAM17) (16). ADAM17 also plays a key role in the proteolytic release from cell membranes of some cytokines including tumor necrosis factor (TNF) α, which is produced by various cell types such as macrophages, monocytes, and T cells, thus playing a central role in the regulation of the immune system and in a variety of inflammatory responses (17). Defects in Klotho expression in mice result in a syndrome that is similar to human aging, including Mönckeberg-type arteriosclerosis from the aorta to small arterioles (13), impairments in angiogenesis and vasculogenesis (6) and endothelial dysfunction (7). Furthermore, *in vivo* gene delivery or upregulation of Klotho expression protect against endothelial dysfunction in experimental models of atherosclerosis (8) and ameliorates vascular calcification in an adenine-induced chronic renal failure model (18, 19). Similarly, endothelial dysfunction in heterozygous Klotho-deficient mice is prevented by parabiosis with wild-type mice (7). Also *in vitro*, Klotho protects vascular smooth muscle and endothelial cells against various stressors (20, 21).

In the clinical scenario, the reduction in circulating Klotho has been proposed as a predictor of atherosclerosis (10) being related with carotid artery intima-media thickness (CIMT) burden, a marker of subclinical atherosclerosis (10, 22), as well as with an increased incidence of cardiovascular disease (CVD) (23). These associations with vascular damage and CVD are particularly relevant in patients with renal disease since Klotho levels are reduced in all stages of the disease (9, 24, 25). Thus, both the reduction of soluble Klotho in blood and the decrease of gene expression in PBCCs have been associated with increased CIMT in patients with moderate to severe chronic kidney disease (CKD) (9, 26, 27). However, the contribution of Klotho expression both in PBCCs and in the carotid artery wall on the CIMT burden in patients with preserved values of estimated glomerular filtration rate (eGFR) has not been assessed yet. Moreover, the relationship of circulating Klotho with the CIMT in patients with preserved kidney function has been refuted by some recent reports (28–30). For all these reasons, we conducted a single-center study in a group of atherosclerotic patients with GFR ≥60mL/min/1.73m^2^ subjected to elective carotid surgery. In this group, we performed different measurements of Klotho protein levels in serum and carotid artery, and of Klotho gene expression in PBCCs and carotids, and analyzed their relationship with CIMT values. We also determined the levels of ADAM-17 and of inflammatory cytokines involved in the atherosclerotic process including tumor necrosis factor α (TNFα) and the interleukins (IL) 6 and 10.

## Patients and methods

### Study design and participants

This cross-sectional single-center proof-of-concept study recruited patients from the Vascular Surgery Service of the University Hospital Nuestra Señora de Candelaria (Santa Cruz de Tenerife, Spain). The study was conducted in accordance with the tenets of the Declaration of Helsinki, and approval was obtained from the local ethics committee. Written informed consent was provided by all participants. Exclusion criteria were estimated eGFR lower than 60 mL/min/1.73m^2^; hemodynamic instability during the surgical procedure (systolic blood pressure (SBP)<90 mmHg and/or administration of inotropes or vasopressors); chronic or acute inflammatory status including: immunologic disease, tumoral disease, hepatitis B, hepatitis C, or HIV, infectious intercurrent episodes in the previous month; receipt of immunotherapy or immunosuppressive therapies; institutionalization; and inability or unwillingness to provide informed consent.

After an initial evaluation of 62 patients, only 35 subjects met the inclusion and exclusion criteria and were finally included in the study. Thus, the study group consisted in 35 patients older than 18 years who underwent elective endarterectomies due to clinical atherosclerotic vascular disease.

At the time of surgery, sections of the affected carotid artery were retrieved. Whole blood samples (3 mL) were also collected in PAXgene Blood RNA tubes (BD, Franklin Lakes, NJ) and in routine blood-collection tubes (BD serum separation transport tube—BD, Franklin Lakes, NJ). Serum fractions were isolated, aliquoted and immediately frozen at -80°C.

Measurements of CIMT were performed by two qualified radiologists who were blinded to the study participant´s details. Ultrasonographies were made with a high-resolution ultrasound (Philips ATL 5000 HDI, Royal Philips Electronics, Amsterdam, The Netherlands).

### Biochemical determinations

The Clinical Analysis Service determined biochemical and hematological parameters by employing standardized tests. Serum levels of Klotho and inflammatory cytokines were measured using commercial ELISA assay kits according to manufacturer’s instructions. For serum Klotho, a solid phase sandwich ELISA (Immuno-Biological Laboratories, Gunma, Japan) – sensitivity: 6.15 pg/mL; intra- and inter-assay coefficients of variation: 2.7–3.5% and 2.9–11.4%, respectively- was employed. Serum levels of cytokines TNFα, IL6 and IL10 were determined with Quantikine® ELISA kits (R&D Systems, Abingdon, UK) -sensitivity: 0.10 pg/mL, 0.70 pg/mL, and 0.09 pg/mL, respectively; intra- and inter-assay coefficients of variability were < 10.8%-.

### Gene expression analysis

Total RNA was extracted from homogenized vascular segments by employing liquid nitrogen with a pestle and mortar and RNAzol RT (Sigma Aldrich, MO, USA). Blood total RNA was isolated using PAXgene Blood RNA Kit (PreAnalytiX, Switzerland). RNA samples were stored at −80°C until being retrotranscribed to cDNA using a High Capacity RNA-to-cDNA kit (Applied Biosystems, CA, USA) for further use in quantitative RT-PCR (qRT-PCR).

Transcripts encoding for Klotho (*KL*), *TNF*, *IL6*, *IL10*, and glyceraldehyde-3-phosphate dehydrogenase (*GAPDH*) genes were measured by TaqMan qRT-PCR employing the following probes: Hs00183100_m1 [*KL*], Hs00174128 m1 [*TNF*], Hs00985639_ml [*IL6*], Hs00961622_m1 [*IL10*], and Hs99999905_m1 [*GAPDH*]. target mRNA was estimated by relative quantification using the 2^-ΔΔCt^ method and *GAPDH* as housekeeping gene. Samples were tested in triplicate and values were expressed as arbitrary units (a.u.).

### Histology and immunohistochemistry

Blood vessels sections were fixed in 4% buffered formalin for 24 h, dehydrated in ascending concentrations of ethanol, cleared in xylene and embedded in paraffin blocks. Trimmed sections of 3µm were processed for histology and immunohistochemistry. Hematoxylin and eosin (HE) staining was performed. For immunohistochemistry, the carotid sections were boiled in sodium citrate solution for 30 min in a Decloaking Chamber™ NxGen (Biocare Medicals, CA, USA) for antigen retrieval and then returned to room temperature. After blocking with 10% Bovine Serum Albumin (BSA) for 30 min. The sections were incubated with 1:100 dilution of rabbit monoclonal anti-Klotho primary antibody (Abcam, ab181373). After washing with PBS, the sections were incubated with the secondary antibody anti-rabbit IgG for 30 min. Following a PBS rinse, sections were developed with 3, 3-diaminobenzidine tetrahydrochloride (DAB) (Sigma-Aldrich, St. Louis, MO, USA) to produce a brown color. For immunoreactivity quantification analysis, images of each slide including intima- media and adventitia layers were captured and processed with a high-resolution video camera (Sony, DF-W-X710, Kōnan, Japan) connected to a light microscope (Nikon Eclipse 50i). Stained brown areas were quantified using ImageJ software (Rasband, W.S., ImageJ, National Institutes of Health, Bethesda, MD, USA). Briefly, Klotho protein expression in the intima-media and adventitia areas was analyzed separately and expressed as square microns (μm^2^) of staining. The immunoreactivity values represent the mean of the quantification values obtained in 5 microscopic fields with the same dimensions that were chosen at random in each stained carotid section.

### Statistical analysis

Normal distribution of continuous variables was checked using the Kolmogorov–Smirnov’s test. Normally distributed variables were expressed as the mean ± SD and non-normally distributed as the median (interquartile range). Categorical data were expressed as number and percent frequency. Differences between groups were analyzed using Chi-square test, Student’s t-test or the Mann–Whitney U-test as appropriate. The Spearman rank correlation test was used to determine bivariate correlations. Backward stepwise multiple regression analysis was performed to determine potential predictor variables (age, sex, body mass index (BMI), hypertension (HT), type 2 diabetes mellitus (DM2), smoking, dyslipidemia, serum uric acid, eGFR, urinary albumin excretion (UAE), phosphorus, serum C-reactive protein (PCR), neutrophil-to-lymphocyte ratio (NLR), serum Klotho, PBCCs and carotid mRNA expression levels of *KL*, *TNF*, *IL6*, and *IL10*, and immunoreactivity for Klotho protein) of CIMT. Collinearity was excluded analyzing the tolerance and variance inflation factor. A value of P < 0.05 was considered to be statistically significant. All analyses were performed using SPSS software version 25 (IBM Corp. Armonk, NY, USA).

## Results

Demographic, clinical and biochemical characteristics of the study population are presented in **Table 1**. A total of 35 patients (27 males), with a mean age of 65 ± 6.9 years, were included in this study. The median and IQR values for CIMT and eGFR were 1.15 (1.0-1.62) mm and 93.05 (82.4-98.2) mL/min/1.73m^2^, respectively. Main comorbidities included DM2 (25.7%), HT (82.9%), smoking (48.6%), and dyslipidemia (45.7%).

**Table 1:** Clinical characteristics and biochemical assessments of the patients included in the study.

	All subjects	HCIMT group	LCIMT group	*p*
Characteristics				
N	35	17	18	
Age (years)	65.7 ± 6.9	67.24 ± 5.6	64.17 ± 7.8	0.189
Sex (% male)	27 (77.1)	15 (88.2)	12 (66.7)	0.132
BMI (kg/m^2^)	28.1 ± 4	28.4 ± 4.7	27.8 ± 3.5	0.611
SBP (mm Hg)	131.7 ± 20.8	134.5 ± 26.3	129.2 ± 14.3	0.474
DBP (mm Hg)	70.31 ± 11.8	74.4 ± 26.3	66.5 ± 11.1	0.047
CIMT (mm)	1.15 (1-1.62)	1.62 (1.31-1.93)	1 (0.94-1.05)	
Comorbidities				
DM2 (%)	9 (25.7)	7 (41.2)	2 (11.11)	0.049
Hypertension (%)	29 (82.9)	15 (88.2)	14 (77.8)	0.358
Current smokers (%)	17 (48.6)	7 (41.2)	10 (55.6)	0.696
Dyslipidemia (%)	16 (45.7)	8 (47.1)	8 (44.4)	0.652
Pharmacological treatment				
Antiaggregants (%)	30 (85.7)	13 (76.4)	15 (83.3)	0.561
Beta-blockers (%)	10 (28.6)	6 (35.3)	4 (22.2)	0.621
ACEI/ARA2 (%)	17 (48.6)	10 (58.8)	7 (38.9)	0.421
CCB (%)	8 (22.9)	3 (17.6)	5 (27.8)	0.264
Statins (%)	31 (88.6)	15 (88.2)	16 (88.9)	0.892
Laboratory data				
eGFR (ml/min/1.73 m^2^)	93.05 (82.4-98.2)	93.05 (88.9-98.7)	90.3 (77.7-96.6)	0.273
Creatinine (mg/dl)	0.84 (0.71-0.91)	0.84 (0.71-0.89)	0.83 (0.71-0.94)	0.708
Albumin (g/dL)	3.9 ± 0.47	3.9 ± 0.47	3.9 ± 0.47	0.966
UAE (mg/g)	23 (1.13-57)	32.3 (18.1-61.2)	9.7 (0.3-49.3)	0.077
Calcium (mg/dL)	9.25 ± 0.41	9.17 ± 0.44	9.3 ± 0.33	0.219
Phosphorus (mg/dL)	3.49 ± 0.45	3.4 ± 0.41	3.58 ± 0.48	0.249
Uric acid (mg/dL)	5.5 ± 1.3	5.8 ± 1.24	5.3 ± 1.34	0.237
Glucose (mg/dL)	103.9 ± 22.7	106 ± 26.3	101.3 ± 19.2	0.502
T-cholesterol (mg/dL)	172.2 ± 49.8	166.6 ± 51	177.4 ± 9.5	0.528
HDL-C (mg/dL)	46.5 ± 11	46.5 ± 12.7	46.5 ± 9.5	0.994
LDL-C (mg/dL)	99.6 ± 46.2	90.9 ± 44.4	107.8 ± 47.7	0.286
TG (mg/dL)	151.4 ± 128	137.7 ± 50.9	164.3 ± 173.2	0.539
Neutrophils (/mL)	5691 ± 2086	5318 ± 1910	6044 ± 2236	0.308
Lymphocytes (/mL)	2199 ± 1058	1834 ± 995	2544 ± 1023	0.045
NLR	3.07 ± 1.55	3.46 ± 1.6	2.7 ± 1.45	0.154
Hb1ac	5.8 (5.2-6.2)	5.8 (5.3-6.4)	5.8 (5.2-6.12)	0.369
hs-CRP (mg/L)	3.6 (0.65-8.6)	3.9 (0.39-9.7)	3.4 (0.95-6.5)	0.708
TNFα (pg/mL)	0.88 (0.67-1.11)	1.04 (0.81-1.32)	0.84 (0.53-0.93)	0.045
IL6 (pg/mL)	0.7 (0.7-1.7)	0.7 (0.7-1.58)	0.7 (0.7-2.6)	0.961
IL10 (pg/mL)	3.9 (0.68-4.92)	3.9 (0.63-5.36)	3.98 (0.67-4.38)	0.757
Klotho (pg/mL)	616.3 (430.8-691)	430.8 (357.7-592.9)	667.8 (632.5-712.9)	<0.001
iFGF23 (pg/mL)	15.62 (9.45-23.9)	15.45 (9.78-23.99)	15.62 (6.41-23.9)	0.884

HCIMT, high carotid-intima thickness; LCIMT, low carotid-intima thickness; BMI, body mass index; SBP, systolic blood pressure; DBP, diastolic blood pressure; CIMT, carotid intima-media thickness; DM2; Type 2 Diabetes mellitus; ACEI/ARA2, Angiotensin converting enzyme inhibitor/angiotensin receptor antagonist 2; CCB, Calcium channels blockers; eGFR, estimated glomerular filtrate rate; UAE, urinary albumin excretion; HDL-C, high-density lipoprotein cholesterol; LDL-C, low-density lipoprotein cholesterol; TG, Triglycerides; NLR, Neutrophil-to-lymphocyte ratio; Hb1ac, glycated hemoglobin; hs-CRP, high sensitivity C-reactive protein; TNFα, tumor necrosis factor; IL, interleukin; KL, Klotho gene; iFGF23, intact form of fibroblast growth factor 23.

**Figure 1:**
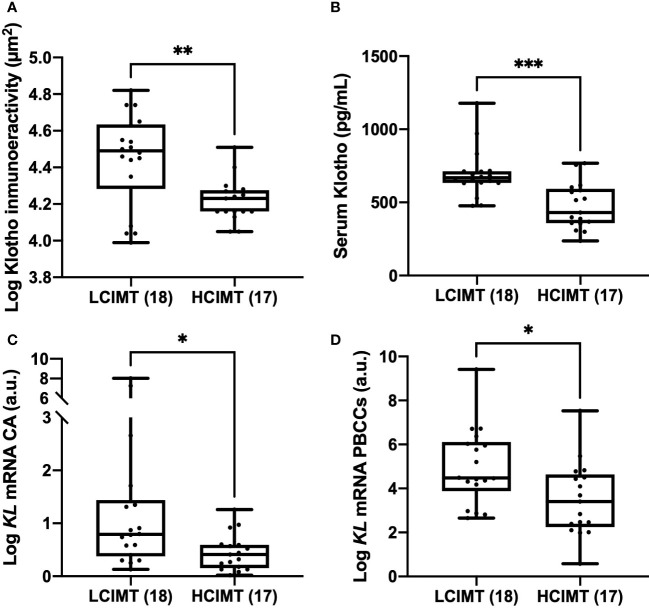
Differences in Klotho vascular immunoreactivity **(A)**, serum levels **(B)**, and mRNA gene expression levels in CA **(C)**, and PBCCs **(D)** according to LCIMT and HCIMT groups. The line within the box represents the median value. The lower and the upper border of the box represent the first and third quartile, respectively. The whiskers indicate maximum and minimum values. HCIMT, high carotid-intima thickness; LCIMT, low carotid-intima thickness; CA, carotid artery; *KL*, Klotho gene; PBCCs, peripheral blood circulating cells. HCIMT, n=17; LCIMT, n=18. *p<0.05; ** p<0.01; ***p<0.001.

The study population was divided into two subgroups according to the median CIMT value: high CIMT (≥ 1.15 mm; HCIMT, n=17) and low CIMT (< 1.15 mm; LCIMT, n=18). The median serum Klotho levels in the HCIMT group were significantly lower than in the LCIMT group (430.8 [357.7-592.9] *vs*. 667.8 [632.5-712.9] pg/mL; p<0.001) (**Table 1**, **Figure 1**). Patients included in the HCIMT group also presented a higher incidence of DM2 (41.2 *vs.* 11.11; p=0.049) and higher levels of serum TNFα (1.04 [0.81-1.32] *vs.* 0.84 [0.53-0.93] pg/mL; p=0.045) (**Table 1**). No differences between groups were observed in the use of antiaggregant, beta-blockers, angiotensin-converting enzyme inhibitor or angiotensin receptor antagonist, calcium channel blockers, or statins.

**Figure 2:**
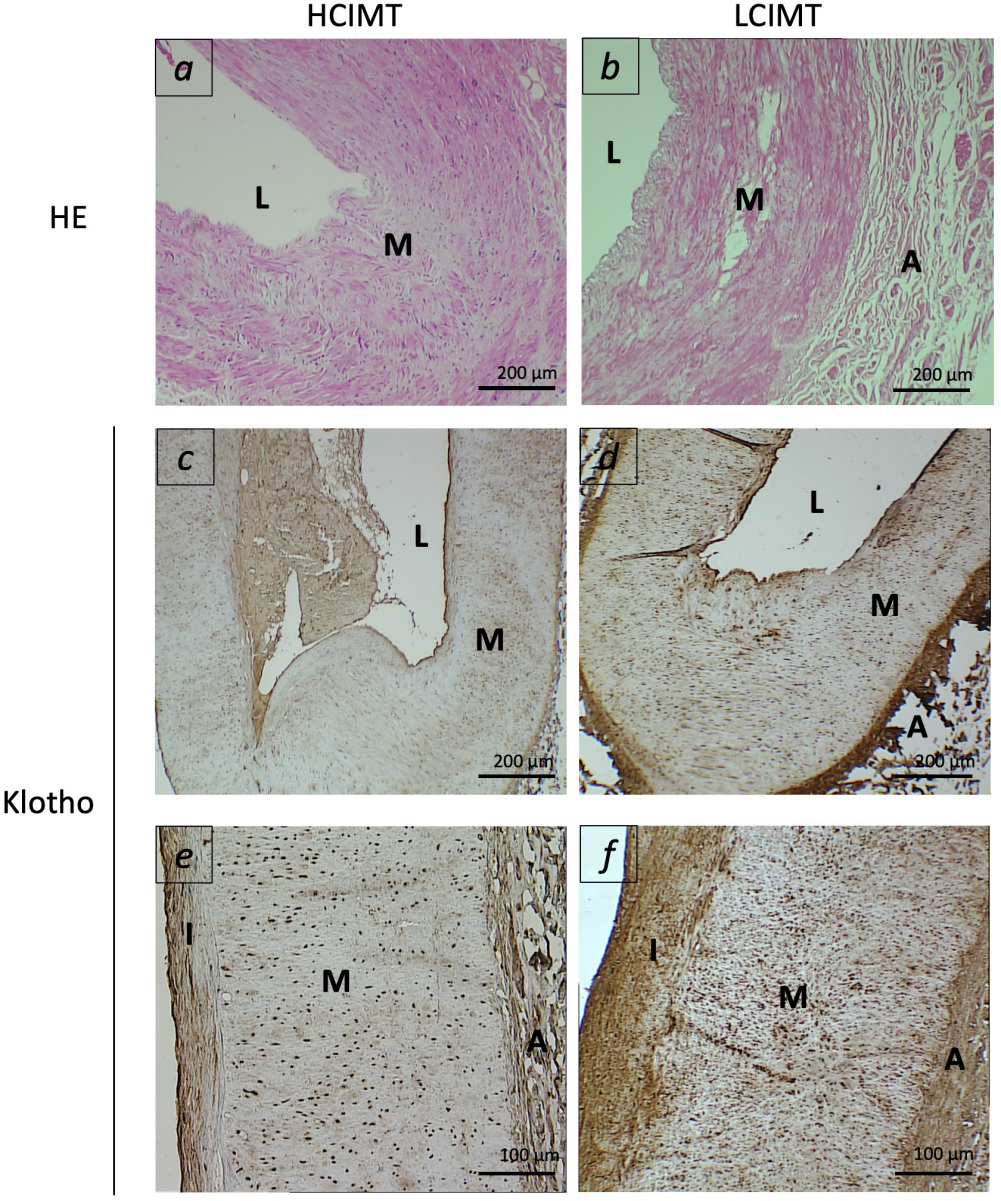
Representative images of carotid arterial sections from patients included in the HCIMT **(A, C, E)** and LCIMT **(B, D, F)** groups. Hematoxylin & eosin (HE) staining **(A, B)** and immunohistochemical staining for Klotho **(C–F)**. HCIMT, n=17; LCIMT, n=18. Original magnification 10x **(A–D)** and 20x **(E, F)**. L, lumen; I, tunica intima; M, tunica media; A, adventitia.

Regarding gene expression, we found reduced mRNA levels of *KL* in the HCIMT group either in PBCCs (2.92 [2.06-4.8] *vs*. 3.69 [2.42-7.13] log a.u., p=0.015) and in carotid artery fragments (0.41 [0.16-0.59] *vs*. 0.79 [0.37-1.4] log a.u., p=0.013) (**Table 2**, **Figure 1**). Klotho protein was detected both in the intimal-medial area and in the adventitia of the carotid sections of all the patients included in the study. No differences were observed in the immunoreactivity of the adventitia according to the subgroups (HCIMT: 3.62 [2.8-4.1] *vs*. LCIMT: 3.71 [2.9-4.3] log µm^2^, p=0.131). However, and similarly to what is observed with *KL* mRNA levels, the immunoreactivity for Klotho protein in the intima-media was reduced in patients included in the HCIMT group (4.23 [4.15-4.27] *vs*. 4.49 [4.28-4.63] log µm^2^, p=0.008) (**Figure 2**). Gene expression of *ADAM17* was also reduced in carotids of HCIMT patients (0.84 [0.61-1.31] *vs*. 1.08 [0.9-2.3] log a.u., p=0.012). Concerning inflammatory cytokines, we only found differences in the expression levels for *TNF* mRNA in PBCCs, which was increased in the HCIMT group (3.73 [1.94-4.12] vs. 2.14 [1.89-2.82] log a.u., p=0.019) (**Table 2**).

**Table 2:** Gene expression levels in PBCCs and CA, and immunoreactivity in CA.

	All subjects	HCIMT group	LCIMT group	*p*
N	35	17	18	
Log PBCCs mRNA (a.u.)				
* TNF*	2.56 (1.89-2.92)	3.73 (1.94-4.12)	2.15 (1.89-2.82)	0.019
* IL6*	2.64 (1.06-5.25)	2.26 (0.69-4.43)	3.1 (1.94-5.64)	0.110
* IL10*	0.35 (0.19-0.74)	0.35 (0.27-0.81)	0.34 (0.16-0.74)	0.351
* ADAM17*	2.14 (1.37-2.56)	2.18 (1.44-2.98)	1.6 (1.25-2.27)	0.11
* KL*	3.03 (2.39-6.1)	2.92 (2.06-4.8)	3.69 (2.42-7.13)	0.015
Log CA mRNA (a.u.)				
* TNF*	1.38 (0.75-2.33)	1.38 (0.7-2.8)	1.38 (0.81-2.1)	0.683
* IL6*	0.52 (0.17-1.39)	0.56 (0.14-1.16)	0.47 (0.17-4.21)	0.423
* IL10*	0.12 (0.04-0.21)	0.14 (0.07-0.18)	0.09 (0.03-0.36)	0.790
* ADAM17*	1.01 (0.78-1.45)	0.84 (0.61-1.31)	1.08 (0.9-2.3)	0.012
* KL*	0.58 (0.27-0.92)	0.41 (0.16-0.59)	0.79 (0.37-1.4)	0.013
Log CA immunoreactivity*				
* KL*	4.3 (4.15-4.5)	4.23 (4.15-4.27)	4.49 (4.28-4.63)	0.008

HCIMT, high carotid-intima thickness; LCIMT, low carotid-intima thickness; PBCCs, peripheral blood circulating cells; TNF, tumor necrosis factor; IL, interleukin; ADAM, A disintegrin and metalloproteinase domain containing protein; KL, Klotho gene; a.u., arbitrary units. *Immunoreactivity in the intimal-medial area.

**Figure 3:**
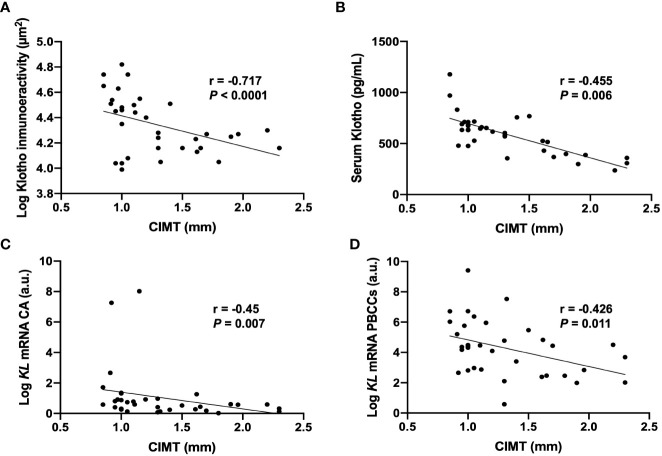
Correlations between CIMT values and Klotho vascular immunoreactivity **(A)**, serum levels **(B)**, and mRNA gene expression levels in CA **(C)** and PBCCs **(D)**. CA, carotid artery; *KL*, Klotho gene; PBCCs, peripheral blood circulating cells. N=35.

Correlation analysis showed that CIMT were inversely correlated with the levels of serum Klotho (r= -0.717, p<0.001), the values of immunoreactivity for Klotho protein in the intima-media area (r= -0.455, *p*= 0.006), and with *KL* mRNA expression levels both in PBCCs (r=-0.426, p=0.011) and in carotid arteries (r= -0.45, p=0.07) (**Table 3**, **Figure 3**). We also found a direct correlation of CIMT values with UAE (r=0.386, p=0.022), and with the PBCCs mRNA expression of *ADAM17* (r=0.419, p=0.012).

**Table 3:** Bivariate correlations between CIMT values and diverse parameters.

	CIMT	
	r	*P*
hs-CRP (mg/L)	-0.013	0.942
TNFα (pg/mL)	0.242	0.162
IL6 (pg/mL)	0.117	0.502
IL10 (pg/mL)	-0.086	0.623
Klotho (pg/mL)	-0.717	<0.001
eGFR (ml/min/1.73 m^2^)	0.098	0.577
UAE (mg/g)	0.386	0.022
T-cholesterol (mg/dL)	-0.177	0.308
HDL-C (mg/dL)	-0.144	0.41
LDL-C (mg/dL)	-0.186	0.283
iFGF23 (pg/mL)	-0.085	0.627
*TNF* mRNA PBCCs (a.u.)	0.239	0.166
*IL10* mRNA PBCCs (a.u.)	0.167	0.339
*IL6* mRNA PBCCs (a.u.)	-0.12	0.491
*KL* mRNA PBCCs (a.u.)	-0.426	0.011
*ADAM17* mRNA PBCCS (a.u.)	0.419	0.012
*TNF* mRNA CA (a.u.)	-0.042	0.815
*IL10* mRNA CA (a.u.)	0.066	0.717
*IL6* mRNA CA (a.u.)	-0.148	0.412
*KL* mRNA CA (a.u.)	-0.450	0.007
*ADAM17* mRNA CA (a.u.)	-0.345	0.047
Klotho CA (µm^2^)*	-0.455	0.006

CIMT, carotid-intima thickness; hs-CRP, high sensitivity C-reactive protein; TNF, tumor necrosis factor; IL, interleukin; eGFR, estimated glomerular filtrate rate; UAE, urinary albumin excretion; HDL-C, high-density lipoprotein cholesterol; LDL-C, low-density lipoprotein cholesterol; iFGF23, intact form of fibroblast growth factor 23; PBCCs, peripheral blood circulating cells; ADAM, A disintegrin and metalloproteinase domain containing protein; CA, carotid artery; a.u., arbitrary units. *Immunoreactivity in the intimal-medial area.

Finally, to test the independent association between Klotho and the CIMT values, we performed a backward stepwise multiple regression analysis. This analysis included CIMT as the dependent variable and diverse potential predictors including, among others, UAE, serum Klotho, PBCCs mRNA expression of *ADAM17*, PBCCs and carotid mRNA expression levels of *KL*, *TNF*, *IL6*, and *IL10*, and immunoreactivity for Klotho protein as covariates. The results showed that only serum Klotho concentrations and mRNA *TNF* expression levels in PBCCs were related with the values of CIMT (adjusted R^2 = ^0.593, p<0.001) (**Table 4**). Tolerance and VIF values were higher than 0.60 and lower than 1.5 for all variables in any of the analysis. Therefore, collinearity was excluded.

**Table 4:** Multiple backward stepwise regression with CIMT value as the dependent variable.

	Adjusted R^2^	ß	Standard error	t	*P*
CIMT (mm)	0.593				<0.001
Klotho (pg/mL)		-0.002	-0.767	-6.56	<0.001
* TNF* mRNA PBCCs (a.u.)		0.105	0.251	2.15	0.04

Covariates included age, sex, BMI, HT, DM2, smoking, dyslipidemia, serum uric acid, eGFR, UAE, phosphorus, PCR, NLR, serum Klotho, PBCCs mRNA expression of *ADAM17*, PBCCs and carotid mRNA expression levels of *KL*, *TNF*, *IL6*, and *IL10*, and Klotho immunoreactivity. CIMT, carotid-intima thickness; TNF, tumor necrosis factor; PBCCs, peripheral blood circulating cells; a.u., arbitrary units.

## Discussion

The results of this study in subjects with eGFR≥60mL/min/1.73m^2^ show that patients with carotid artery atherosclerosis and higher values of CIMT present reduced soluble Klotho levels, as well as decreased *KL* mRNA expression in PBCCs and Klotho protein levels in the intima-media of the carotid artery wall. In multivariate analysis, only serum Klotho was found to be an independent determinant of CIMT value, together with *TNF* expression in PBCCs. Several clinical and preclinical studies have suggested that Klotho exerts strong vascular protective effects. For instance, higher soluble Klotho levels have been related to a lower mortality and CVD morbidity (23, 31, 32). Conversely, in patients with CKD, a condition characterized by reductions of Klotho protein from early stages, low circulating Klotho concentrations have been associated with increased arterial stiffness and CIMT (9, 26). However, studies about the relationship between Klotho and CIMT in patients with atherosclerosis and preserved kidney function are scarce and none of them had previously considered the expression levels of Klotho in PBCCs and atherosclerotic vascular tissue.

Circulating Klotho may participate in vascular health by protecting against endothelial dysfunction (7, 8) which plays an important role in the development of atherosclerosis (33). But beyond its soluble form, potential benefits of Klotho in vascular homeostasis may also come from its expression in vascular tissue and in PBCCs. Klotho is endogenously expressed in human vascular smooth muscle cells (14, 32) and although its exact role in the vasculature remains to be elucidated, inhibition of Klotho expression in these cells results in accelerated calcification (32). Moreover, its reduction is associated with coronary artery disease (31) and is inversely related with inflammation in atherosclerosis (11). Bivariate analyses presented in this work show inverse relationships of CIMT with vascular Klotho, both at mRNA and protein levels. However, after adjusting for age, gender, blood pressure, smoking, DM2, dyslipidemia, albuminuria and other cardiovascular risk factors and inflammatory markers, only the inverse association of serum Klotho with CIMT remained as statistically significant.

The association between low Klotho and CVD has been recently extended to PBCCs, in which reductions in Klotho expression have been related with aging process and with the development of pathologies with an inflammatory component, including atherosclerosis (26, 34–36). Macrophages, monocytes, lymphocytes, and other PBCCs play an important role in development of the inflammatory response associated with atherogenesis (37, 38). Upregulation of Klotho expression in PBCC could have beneficial anti-inflammatory actions leading to better progression of atherosclerotic lesions. Thus, Klotho expression in PBCCs has been related with the attenuation of acute inflammation induced by lipopolysaccharides, with the inactivation of NF-kB signaling and with the promotion of M2 polarization in macrophages (39). Likewise, the stimulation of Klotho expression in these cells has also been related with the suppression of the stress response of the Golgi apparatus and endoplasmic reticulum, the reduction of oxidative stress and pro-inflammatory cytokines production, and with an increased expression of anti-inflammatory cytokines as well as with the preservation of the immune function in senescent monocytes (40, 41). Moreover, we recently reported an association between Klotho expression in PBCCs and vascular and systemic inflammation in atherosclerotic vascular disease (15) and with subclinical atherosclerosis in CKD patients (26).

Besides transmembrane Klotho shedding, ADAM17 is responsible of proteolytical cleavage of several cell surface proteins highly expressed in leukocytes which are involved in the inflammatory response, such as TNFα, TNF-RI, TNF-RII, IL-6R, RANKL, INF-γ or CD40 (42). As shown in **Tables 1** and **2**, patients in the HCIMT group showed a steepen pro-inflammatory state marked by a tendency to have higher values of neutrophil-to-lymphocyte ratio and significant higher levels of serum TNF-α or *TNF* gene expression. So, a higher expression of ADAM17 in PBCCs of these patients could respond to the need to exert a pro-inflammatory response by leukocytes in the context of atherosclerotic damage. In the case of the vascular wall, several cytokines, receptors, adhesion molecules or growth factors expressed in this tissue, and directly related to the setting of atherosclerosis, are substrates of ADAM17. Actually, ADAM17 is considered a pro-inflammatory molecule in the context of atherosclerotic damage, since it is capable to mediate endothelial damage, vascular inflammation, and to promote plaque vulnerability. In fact, several studies indicate that overexpression of ADAM17 is linked to exacerbation of the atherosclerotic process (43–45). Surprisingly, we observed that the expression of this metalloproteinase in the HCIMT group in CA is reduced compared to individuals with LCIMT in our cohort, which is an unexpected finding that might be related to the low sample size of the study.

Although this work provides novel information about the relationship between Klotho and atherosclerosis, we acknowledge several limitations. This is a proof-of-concept study, where the main limitation is the small sample size and the cross-sectional design, which only allowed us to show associations without definitive inferences about their direction or causality. Moreover, our results only show relationships in an already developed clinical scenario and do not provide information about the independent implication of reductions in Klotho levels with the development and progression of the carotid artery atherosclerotic lesion. In addition, we did not measure ADAM10 expression values neither in PBCCs nor in arterial tissue. This alpha-secretase is also responsible of the shedding of Klotho and, similarly to ADAM17, is known to cleave inflammatory mediators, including TNFα. Moreover, we did not analyze the effect of confounders other than serum Klotho levels as potential predictors of atherosclerosis Specifically, UAE has been previously related with subclinical atherosclerosis (46), so we cannot exclude the contribution of albuminuria to CIMT values in our study.

Moreover, the expression of Klotho is also present in the adventitia, probably in the fibroblasts (47). Although we did not find differences between groups in the immunoreactivity for Klotho in this layer, the results of the gene expression study depicted in this work did not differentiate between intima-media and adventitia, so we cannot exclude that the differences in mRNA expression observed might be due to differential expression of this protein in the different layers of the carotid artery.

Given the nature of this study, carried out in normal clinical practice conditions and with patients undergoing elective surgery, we were unable to have a control group. In order to partially solve this limitation, and although it is not the ideal situation, we have used the group of patients with low CIMT in the comparative analysis. Finally, the small sample size that could make that some of our results did not reach statistical significance and, consequently, the ability to describe the association between some variables including vascular and PBCCs expression of Klotho might be limited. Also, this limitation could make that the findings made in this group not be transferable to larger populations.

Nevertheless, our study presents some strengths. Together with serum, we have also determined Klotho expression levels in PBCCs and in vascular tissue, which have a clear implication with the progression of atherosclerosis. Moreover, data included conventional and CVD-related cardiovascular risk factors, including the serum, PBCCs and vascular expression of ADAM-17 and of inflammatory cytokines involved in the atherosclerotic process.

In conclusion, we found that lower Klotho levels are inversely correlated with CIMT values in a group of patients with atherosclerosis and preserved kidney function. Our results are in line with previous findings in the literature that point to soluble Klotho as a protective factor against cardiovascular disorders. Further experimental studies are needed to delve into the interactions between Klotho and the progression of the atherosclerosis burden. In particular, *in vitro* studies are needed to determine the underlying mechanisms by which Klotho is able to maintain endothelial integrity and prevent vascular smooth muscle cell differentiation into proatherogenic phenotypes. These effects may be mediated by reducing oxidative stress and inflammation, and by maintaining endothelial nitric oxide production.

## Data availability statement

The raw data supporting the conclusions of this article will be made available by the authors, without undue reservation.

## Ethics statement

The studies involving human participants were reviewed and approved by Comité Ético de Investigación con Medicamentos (CEIm). The patients/participants provided their written informed consent to participate in this study.

## Author contributions

JD-C: funding acquisition, writing—original draft preparation, acquisition, analysis, and interpretation of data, manuscript submission. EM-N: patient inclusion, acquisition, analysis, and interpretation of data. AM-O and VT: acquisition, analysis, and interpretation of data. CF: acquisition of data; ÁL-C, AD-M, VL-T, MA-G, NP-D, and AG-L acquisition of data and revising the manuscript. CM-F and JN-G: funding acquisition, design and planning of the work, patient inclusion, acquisition, analysis, and interpretation of data, and drafting the work. All authors have read and agreed to the published version of the manuscript.
